# Abnormal Bedside Ultrasound Findings in a Complex Patient With Kawasaki Disease

**DOI:** 10.7759/cureus.17112

**Published:** 2021-08-11

**Authors:** Dipal Shah, Helene Koumans, Kimberly Johnson, Leoh N Leon, Latha Ganti

**Affiliations:** 1 Emergency Medicine, Ocala Regional Medical Center, Ocala, USA; 2 Emergency Medicine, Brown University, Providence, USA; 3 Emergency Medicine, Osceola Regional Medical Center, Orlando, USA; 4 Emergency Medicine, Envision Physician Services, Plantation, USA; 5 Emergency Medicine, University of Central Florida College of Medicine, Orlando, USA; 6 Emergency Medicine, HCA Healthcare Graduate Medical Education Consortium Emergency Medicine Residency Program of Greater Orlando, Orlando, USA

**Keywords:** kawasaki disease, focused assessment with sonography for trauma, abdominal pain, pocus (point of care ultrasound, epigastric pain

## Abstract

We herein report a case of an 18-year-old female with Kawasaki disease who presented to the emergency department with epigastric abdominal pain and was subsequently found to have free fluid present in her abdomen visualized on bedside Focused Assessment with Sonography for Trauma (FAST) exam. Kawasaki disease is an acute vasculitis syndrome that primarily affects children and can have serious complications such as coronary artery aneurysms. The use of ultrasound in emergency departments is rapidly increasing, with the FAST being one of the most commonly performed bedside ultrasound examinations. FAST exams are most commonly performed in trauma patients as well as being part of the Advanced Trauma Life Support (ATLS) protocol. However, this case demonstrated that the FAST exam can also have application in other clinical scenarios and patient presentations where there is clinical suspicion of free intra-abdominal fluid.

## Introduction

Kawasaki disease (KD), also termed mucocutaneous lymph node syndrome, is an acute vasculitis syndrome primarily affecting small to medium-sized arteries of children under five years old. Although the etiology is unknown, patients with KD frequently present with fever, swelling of the hands and feet, rash, swollen glands in the neck, and irritation of the mouth, lips, throat, and eyes.

KD causes inflammation in artery walls, and serious complications include coronary artery dilations and aneurysms (CAA). KD is a leading cause of acquired heart disease in the United States and the leading cause of pediatric ischemic heart disease, though a standard treatment of intravenous immunoglobulin and aspirin has been developed to stifle the development of coronary artery abnormalities [1].

Mild dilation or ectasia of the coronary arteries occurs in up to approximately 40% of patients, and coronary artery aneurysms occur in 15-25% of untreated patients. The standard treatment of intravenous immunoglobulin and aspirin, an anti-platelet medication, significantly decrease the development of coronary artery complications. Additionally, coronary artery bypass grafting (CABG) has proven safe and effective for KD-associated coronary heart disease.

The subsequent report describes a case of a patient presenting with epigastric pain and significant free fluid upon conducting a Focused Assessment with Sonography for Trauma (FAST) scan, which assesses the pericardium and three spaces within the peritoneal cavity for fluid, and has proven to be quite sensitive. The symptoms of the case were of particular note due to the history of KD, CAA, and CABG.

## Case presentation

An 18-year-old female with history of KD, coronary artery aneurysms, and coronary artery bypass grafting (CABG) presents to the emergency department by EMS reporting abdominal pain starting earlier this morning. The patient describes the abdominal pain as epigastric radiating to her right shoulder and worsened with deep inspiration. The pain has been worsening throughout the day. She also endorses nausea. She denies vaginal bleeding and reports that her last menstrual period was approximately one month ago. She endorses a history of bulimia, and reports forceful vomiting episodes every day for the past year. Otherwise, the patient denies fever, cough, recent illness, trauma or injury. She notably has a history of KD as a child resulting in coronary artery aneurysms and requiring CABG surgery at age 12. She is currently taking warfarin and aspirin.

Physical exam is remarkable for blood pressure of 99/69 mmHg and heart rate of 98 beats per minute, remaining vital signs within normal limits. On exam, the patient is in no acute distress. The exam is significant for diffuse abdominal tenderness with voluntary guarding.

**Figure 1:**
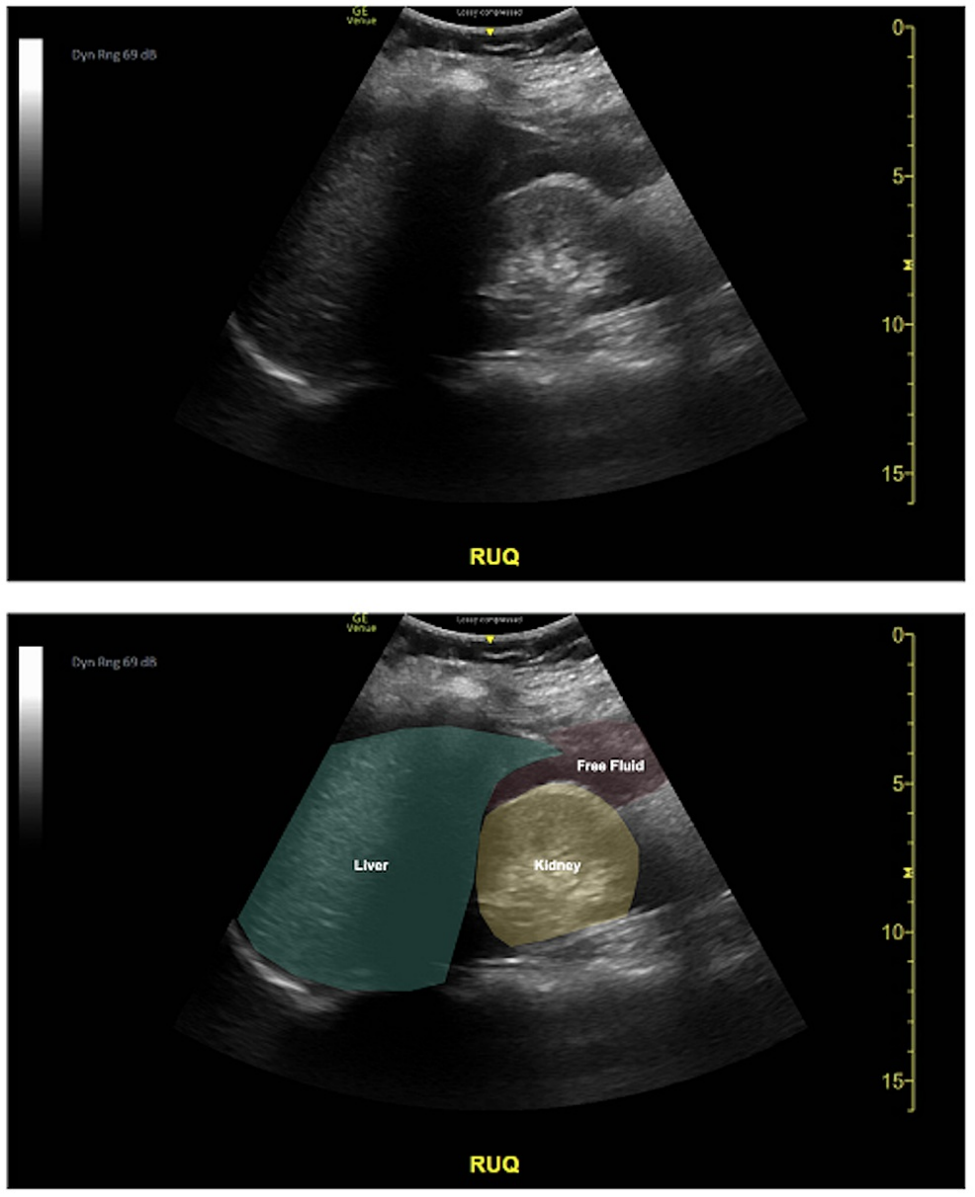
Right upper quadrant view seen on bedside ultrasonography. Transducer was placed at the mix-axillary line at approximately the 10th rib with the probe indicator towards the patient's head visualizing the diaphragm, liver, kidney and a potential space known as “Morrison’s Pouch.” This image demonstrates fluid, visualized as an anechoic (black) area inside of Morrison’s Pouch. The lower image is a color-coded version of the original image for visual interpretation.

**Figure 2:**
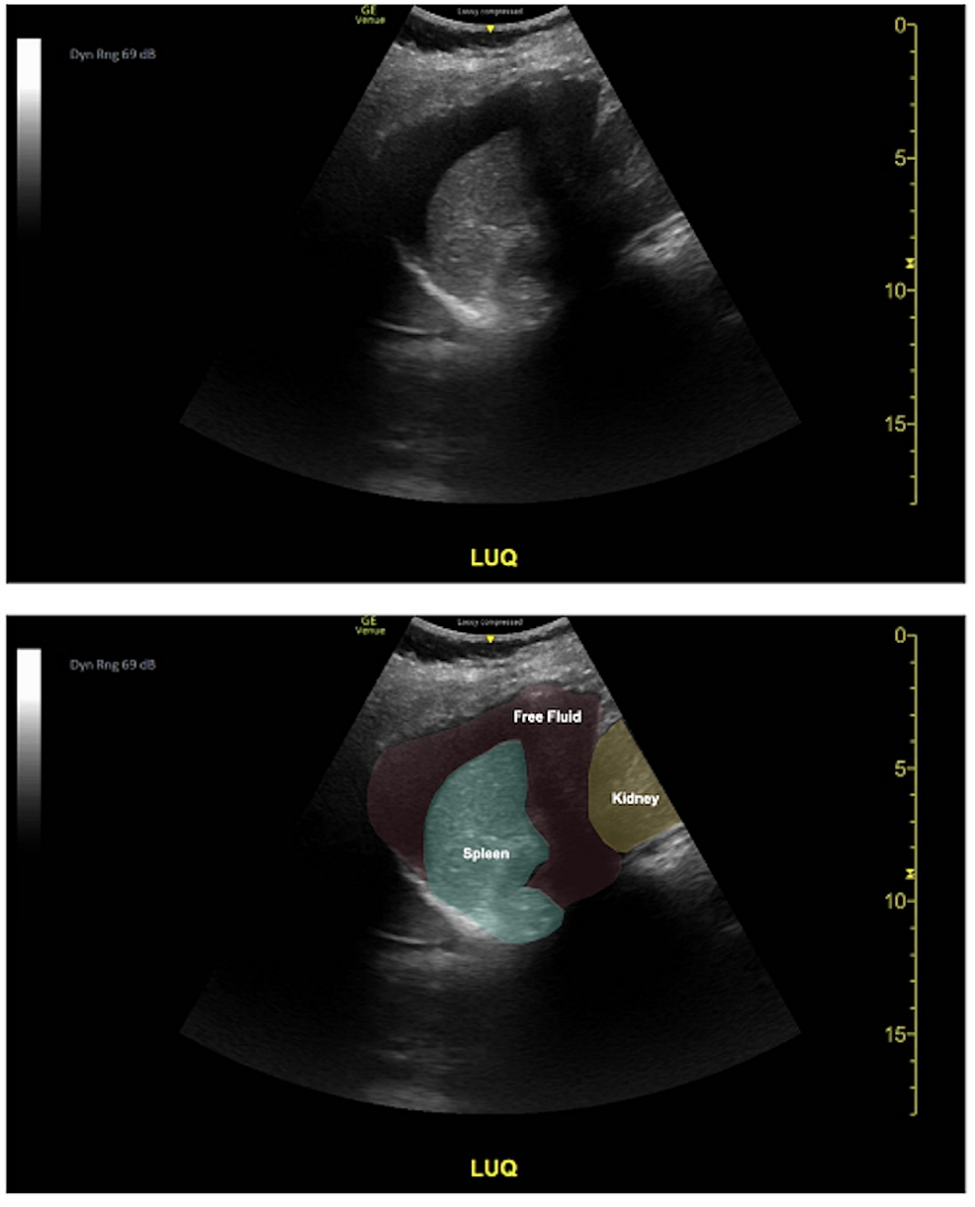
Left upper quadrant view seen on bedside ultrasonography. To obtain this view, the transducer is placed on the posterior axillary line at approximately the 8th rib as the spleen is more posterior and superior in relation to the liver. This image is similar to that of the RUQ and demonstrates the diaphragm, spleen, kidney. In this image we appreciate an anechoic (black) area between the spleen and kidney as well as superiorly to the spleen. The lower image is a color-coded version of the original image for visual interpretation.

Due to significant abdominal tenderness on initial physical exam, bedside FAST scan was performed, which was found to be positive, demonstrating a significant amount of free fluid in the right upper quadrant, left upper quadrant, and suprapubic views (Figures 1, 2).

**Figure 3:**
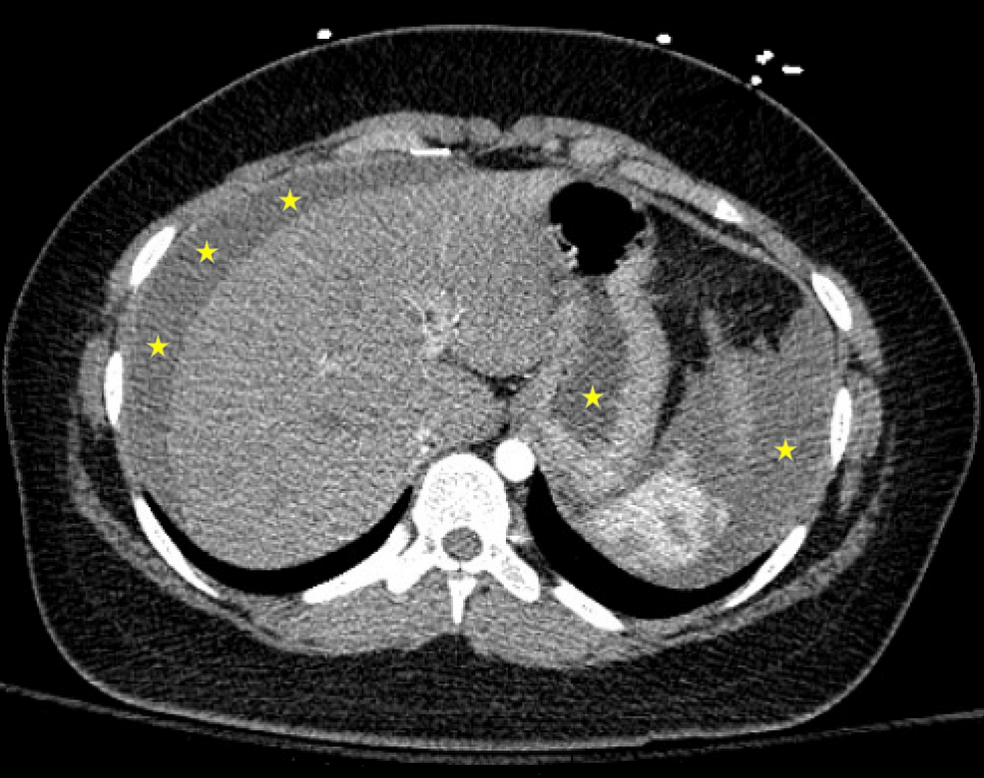
Non-contrast computed tomography (CT) scan demonstrating free fluid (asterisks).

Other workup was remarkable for anemia with a hemoglobin of 8.0 g/dl and negative pregnancy test. Surgery was consulted and computed tomography angiography (CTA) of the chest, abdomen, pelvis were performed emergently due to concern for internal bleeding. CTA imaging demonstrated no acute findings of the aorta, and coronary artery aneurysms at the right coronary artery (RCA) and left anterior descending artery (LAD). Non-contrast CT of the abdomen and pelvis demonstrated free fluid (Figure 3).

Prothrombin complex concentrate (PCC) and tranexamic acid (TXA) were given due to concern for bleeding on anticoagulation. Due to ongoing concern for internal bleeding along with the patient's risk factors, the patient was admitted to the surgical intensive care unit (ICU) for further management.

In the ICU, the patient was transfused 2 units of packed red blood cells. Serial hemoglobin checks and abdominal exams were performed with a plan for diagnostic laparoscopy if hemoglobin decreased. However, the patient’s hemoglobin remained stable, abdominal pain improved, and the patient was eventually discharged to home the following day with close follow-up.

## Discussion

**Figure 4:**
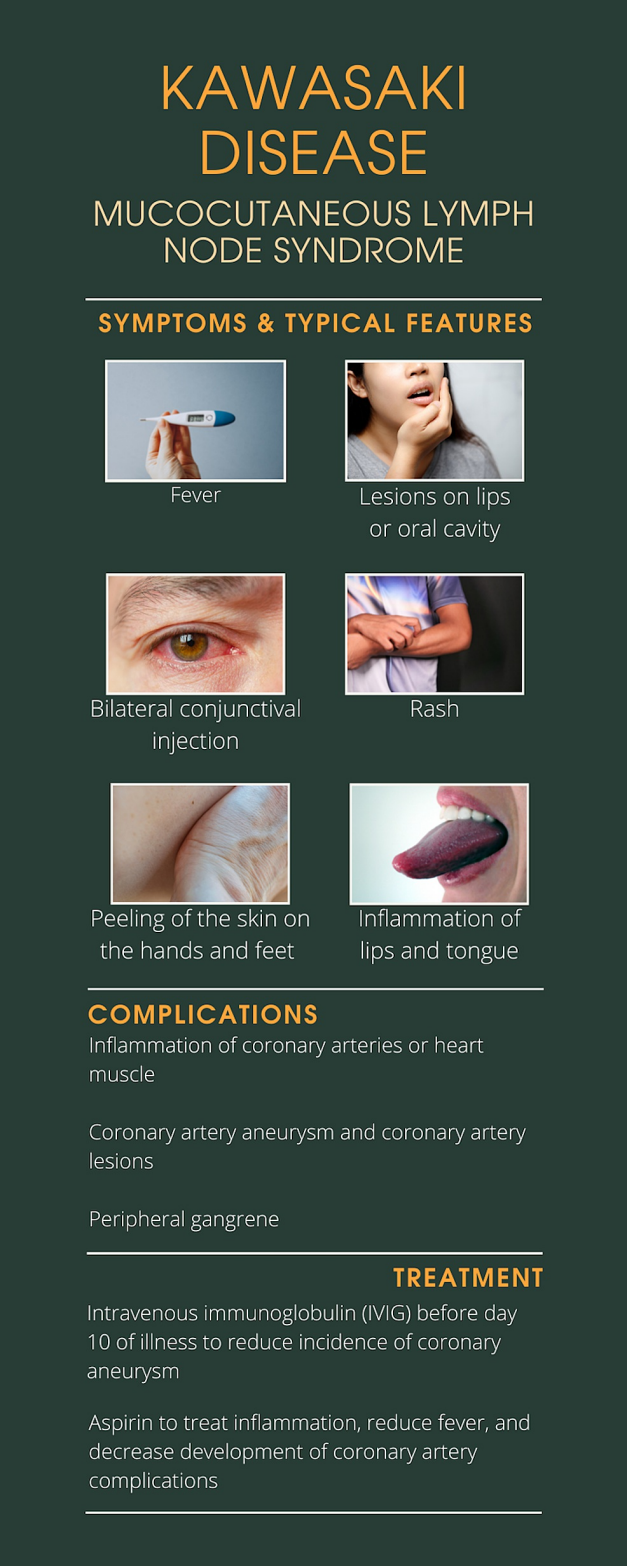
Infographic depicting typical features of Kawasaki disease. Designed by Helene Koumans on Canva.

The case presented with abdominal pain and a history of pediatric KD resulting in coronary artery aneurysms (Figure 4). The patient was already taking warfarin and aspirin. Aspirin is a standard treatment for KD [2-4], and there is ongoing inquiry into the safety and efficacy of warfarin as an accompanying treatment [5,6].

In addition to the patient’s initial physical exam, a bedside FAST scan was emergently performed upon the patient's arrival via ambulance, after which a CTA was performed out of concern for internal hemorrhage, which may occur in KD patients secondary to a ruptured aneurysm. The FAST scan indicated a significant amount of free fluid, and prothrombin complex concentrate (PCC) and tranexamic acid (TXA) were administered due to concern for internal bleeding in a patient on anticoagulants. At present, there has not been considerable investigation of the use of FAST scans in cases of KD or those with history of KD. Further research has been conducted in adjacent fields as a result of the KD-like symptoms of the rare multisystem inflammatory syndrome in children reported as a post-infectious complication of severe acute respiratory syndrome coronavirus 2 (SARS-CoV-2), now termed pediatric inflammatory multisystem syndrome temporally associated with SARS-CoV-2 (PIMS-TS) [7].

FAST scans have demonstrated sensitivities between 85% and 96% and specificities above 98%. Additionally, FAST scans can be conducted in fewer than five minutes and decrease time to surgical intervention [8]. Further study should be done of the benefits and risks of FAST scans in cases presenting abdominal pain and with a history of KD and coronary artery aneurysm. In this case, the FAST scan was critical in the identification of significant free fluid, which allowed for expedient follow-up laboratory work and treatment. Additional study of FAST scans with similar case presentations should be conducted to determine follow-up interventions necessary.

## Conclusions

Prompt identification of free fluid and possible internal hemorrhage is critical in timely administration of follow-up treatment in the emergency department. Untreated internal bleeding, which may be exhibited in KD patients or patients with a history of KD, may result in organ failure, seizure, coma, and death. The performance of a FAST scan allowed for expedient identification of free fluid, subsequently allowing for necessary laboratory work and treatment. Thus, particularly among patients with a history of KD and presenting with abdominal pain, further study should be conducted on the performance of FAST scans in the determination of necessary intervention.
